# Dataset of quality assurance measurements of rhythmic movements

**DOI:** 10.1016/j.dib.2023.109556

**Published:** 2023-09-09

**Authors:** Liran Ziegelman, Tanvi Kosuri, Husain Hakim, Luqi Zhao, Abdelwahab Elshourbagy, Kelly Alexander Mills, Timothy Patrick Harrigan, Manuel Enrique Hernandez, James Robert Brašić

**Affiliations:** a Department of Kinesiology and Community Health, University of Illinois at Urbana-Champaign, Urbana, IL 60801, United States; b Department of Public Health Studies, Krieger School of Arts and Sciences, The Johns Hopkins University, Baltimore, MD 21218, United States; c Section of High-Resolution Brain Positron Emission Tomography Imaging, Division of Nuclear Medicine and Molecular Imaging, The Russell H. Morgan Department of Radiology and Radiological Science, School of Medicine, The Johns Hopkins University, Baltimore, MD 21205, United States; d Misr University for Science and Technology, Al Motamayez District-6th of October, Giza Governorate 3236101, Egypt; e Department of Neurology, School of Medicine, The Johns Hopkins University, Baltimore, MD 21205, United States; f Research and Exploratory Development, Applied Physics Laboratory, The Johns Hopkins University, Laurel, MD 20723, United States; g Department of Behavioral Health, New York City Health + Hospitals/Bellevue, 462 First Avenue, New York, NY 10016, United States; h Department of Psychiatry, New York University Grossman School of Medicine, 550 First Avenue, New York, NY 10016, United States

**Keywords:** Accelerometer, Clinical trial, Continuous wavelet analysis, Fourier analysis, Movement disorders, Typical development, Value proposition, Wearable sensors

## Abstract

A low-cost quantitative structured office measurement of movements in the extremities of people with Parkinson's disease [1,2] was performed on participants with Parkinson's disease and multiple system atrophy as well as age- and sex-matched healthy participants with typical development. Participants underwent twelve videotaped procedures rated by a trained examiner while connected to four accelerometers [1,2] generating a trace of the three location dimensions expressed as spreadsheets [3,4].

The signals of the five repetitive motion items (3.4 Finger tapping, 3.5 Hand movements, 3.6 Pronation-supination movements of hands, 3.7 Toe tapping, and 3.8 Leg agility) [1] underwent processing to fast Fourier [5] and amor and bump continuous wavelet transforms [6], [7], [8], [9], [10], [11], [12], [13].

Images of the signals and their transforms [4], [5], [6] of the five repetitive tasks of each participant were randomly expressed as panels on an electronic framework for rating by 35 trained examiners who did not know the source of the original output [14]. The team of international raters completed ratings of the signals and their transforms independently using criteria like the scoring systems for live assessments of movements in human participants [1,2]. The raters scored signals and transforms for deficits in the sustained performance of rhythmic movements (interruptions, slowing, and amplitude decrements) often observed in people with Parkinson's disease [15], [16], [17], [18], [19], [20].

Raters were first presented the images of the signals and transforms of a man with multiple system atrophy as a test and a retest in a different random order. After the raters completed the assessments of the man with multiple system atrophy, they were presented random test and retest panels of the images of signals and transforms of ten participants with Parkinson's disease who completed a single rating session. After the raters completed the assessments of the participants with Parkinson's disease who completed one set of ratings, they were presented random test and retest panels of the images of signals and transforms of (A) ten participants with Parkinson's disease and (B) eight age- and sex-match healthy participants with typical development who completed two rating session separated by a month or more [15], [16], [17], [18], [19], [20].

The data provide a framework for further analysis of the acquired information. Additionally, the data provide a template for the construction of electronic frameworks for the remote analysis by trained raters of signals and transforms of rhythmic processes to verify that the systems are operating smoothly without interruptions or changes in frequency and amplitude. Thus, the data provide the foundations to construct electronic frameworks for the virtual quality assurance of a vast spectrum of rhythmic processes. The dataset is a suitable template for solving unsupervised and supervised machine learning algorithms. Readers may utilize this procedure to assure the quality of rhythmic processes by confirming the absence of deviations in rate and rhythm. Thus, this procedure provides the means to confirm the quality of the vast spectrum of rhythmic processes.

## Graphical abstract

**Figure:**
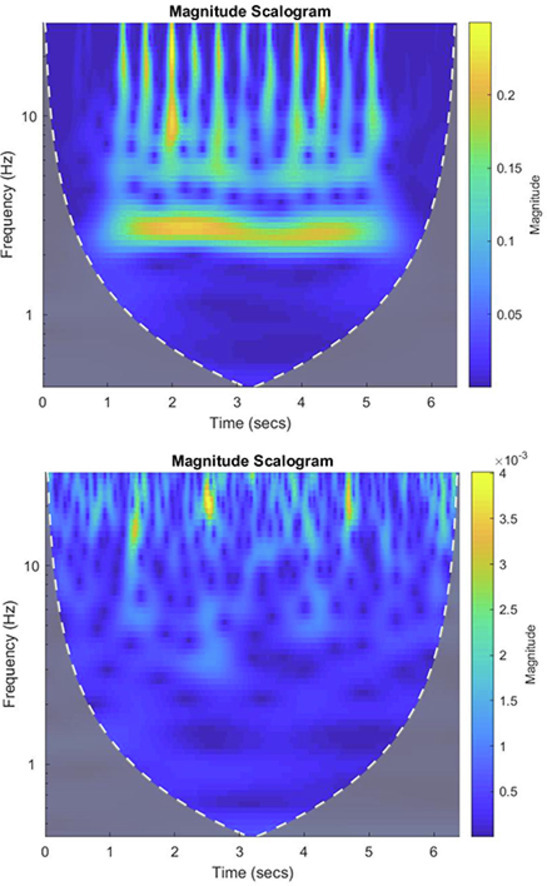

Specifications Table

**Table:** 

Subject	Health and medical sciences
Specific subject area	Accelerometry Assessment Clinical neurology Engineering Extrapyramidal disorders Instrumentation Mathematical modeling Motor evaluation Movement disorders Parkinson's disease
Type of data	Image Video
How the data were acquired	Hardware: • Dell Latitude (E6530) premier laptop [7] • Small, low power, 3-axis ± 3 g accelerometer, ADXL335 [8] • Three-axis accelerometer evaluation board, EVAL-ADXL335Z [9] • USB/Ethernet data logger system [10] • USBMAX, six-foot high-speed USB revision 2.0 shielded MSL cable, 28A WG/2C+24AWG/2C (UL) E305688 type CM 75°C CSA 204790 [13] • Woods 55213143 16/2 low voltage lighting cable, 100-feet [11] Software: • Excel [4] • WinDaq [3] • Continuous wavelet transform [6] • Fast Fourier transform [5] • R [14]
Data format	Raw Analyzed
Description of data collection	A low-cost quantitative measurement of movements in the extremities [1,2] was administered to participants with Parkinson's disease, multiple system atrophy, and typical development. Signals from extremity accelerometers and their fast Fourier [5] and continuous wavelet [6] transforms [12,13,15,16] were randomly represented as panels on an electronic database [15], [16], [17], [18], [19], [20] to be scored for alterations in frequency and amplitude by trained raters unfamiliar with the sources of the images. The images include the original signals from the accelerometers and their fast Fourier [5] and amor and bump continuous wavelet [6] transforms.
Data source location	Institution: Johns Hopkins University City/Town/Region: Baltimore/Maryland Country: United States of America Latitude: 9-17’19” N (39.2970515° N) Longitude: 076-35’30” W (-76.591633° W)
Data accessibility	Repository name: Mendeley Data Data identification number: 8 Direct URL to data: https://doi.org/10.17632/fjxt6cjptn.8[16]
Related research article	M.H. Hernandez, L. Ziegelman, T. Kosuri, H. Hakim, L. Zhao, K.A. Mills, J.R. Brašić, Classification of extremity movements by visual observation of signals and their transforms, MethodsX 9 (2022) 101739. https://doi.org/10.1016/j.mex.2022.101739[17]

## 1 Value of the Data

• These data provide a template for the virtual assessment by trained raters of the key components of the fundamental rhythm of a vast spectrum of regular processes by means of evaluation of output signals and their transforms randomly represented in panels for scoring by raters who lack knowledge of the origin of the signals and transforms.
• Technologists, administrators, clinicians, and others who need to verify the uniformity of rhythmic processes in a variety of settings will benefit from these data to utilize as a framework to assure the quality of essential rhythmic processes.
• Quality assurance officers who require continuous rhythmic processes will benefit from these data as a template (A) to identify interruptions, slowing, and amplitude decrements of crucial processes that require rhythmic performance for optimal function and (B) for structured virtual assessments by trained raters in remote locations.
• Neurologists and neuroscientists seeking optimal assessments of people with Parkinson‘s disease and other disorders will utilize these data for clinical and research purposes.
• These data will serve as a template to be modified for the reliable and valid virtual quality assurance of processes that require rhythmic performances without interruptions, slowing, or amplitude decrements.
• These data will provide the basis for the development of techniques for the precise and accurate assessment of the regularity of rhythmic processes in distant locations.
• The research community will utilize these data as templates for the assessment of the quality of their own datasets of rhythmic processes.

## 2 Objective

Optimal functioning of many processes and services depends on the regular performances of rhythms set at a uniform rate. Quality assurance of the performances of activities without interruptions, halts, slowing, and amplitude decrements is crucial to the maintenance of the products and services as required. Verification of the regularity of the underlying processes is vital to the maintenance of optimal functioning.

Confirming that rhythmic processes are proceeding as required to perform the needed tasks may require the visual inspection of the actual processes and machinery. However, visual observation of the actual processed by qualified viewers may require going to dangerous, inconvenient locations. Therefore, the representation of rhythmic movements by continuous quantitative signals provides a means to assess the performances of rhythmic processes without the limitations inherent in visual inspection by qualified human inspectors.

For example, the representation of movements in the extremities by the output of accelerometers provided the basis to develop a system to assess whether or not the desired repetitive movements were performed at a uniform rhythm without halts, interruptions, or alterations of frequency or amplitude. This example provided the foundation for the development of electronic frameworks for the signals of the accelerometers and their transforms can be represented as images for blind rating for deficits by trained experts who are unfamiliar with the source of the outputs. This electronic framework provides a template for quality assurance of optimal functioning of machinery and other services/products required by multiple industries and fields of endeavor.

Although this dataset was obtained to identify deficits in the performance of rhythmic processes by humans, the general framework also constitutes the basis for the quality assurance of rhythmic processes by machinery and technology utilizing a vast spectrum of rhythmic activities. The sequential publications of the procedure dealt with the measurement of movements by humans conducting repetitive tasks [1,12,17,21]. The goal of the current manuscript is to provide the means to generalize these data for worldwide use to assess the qualities of general rhythmic processes. Because of the great value of these data to enhance the performance of measurements of products/services requiring rhythmic processes, we seek to provide this preliminary report about this dataset to colleagues around the world as a guide to assure the quality of required activities.

We assessed the reliability and validity of a set of signals generated in Baltimore, Maryland, United States, by the administration of a low-cost quantitative measurement of movements in the extremities [1,2] to participants with Parkinson's disease, multiple system atrophy, and typical development. We obtained the independent ratings for interruptions, slowing, and amplitude decrements of 35 trained experts who were unfamiliar with the origin of the signals and transforms and who were located throughout the world.

An electronic framework was constructed in Urbana, Illinois, United States, to represent the signals from the accelerometers attached to the extremities of participants in Baltimore, Maryland, United States, and their transforms so that international raters could access the images and submit rating scores to the administrator for future analysis [15], [16], [17]. The electronic framework provided the means for 35 trained rates around the world to independently visualize and score the random representation of accelerometer signals and their fast Fourier [5] and amor and bump continuous wavelet [6] transforms. The scores were then analyzed, stored, interpreted by technologists at the central base in Urbana [15.16].

## 3 Data Description

This dataset represents a portion of the data acquired by the authors through a series of investigations commencing in 2016 [1,12,17,21] to modify and enhance the gold-standard tool for the motor assessment of people with Parkinson's disease [22]. In this article we explain the details of data collection and validation for the dataset of this article and the related datasets [Table 1]. Table 1 represents a description of the many datasets generated for this article. The relations among the many components of this project are represented to facilitate organizing the materials by readers. The same identification numbers have been utilized through all folders, files, and documents.

**Table 1:** Characterization of the datasets. The content of the many datasets utilized in the projects described in the article are described in detail. Since the same identification numbers are utilized throughout all materials, the interrelationships among the items can be constructed with the use of this table.

Citation	Type of data	Description of files
McKay et al*.,* [1]	Written protocol	Initial attempts of investigators to construct a protocol to utilize instrumentation to generate a low-cost quantitative continuous measurement of movements in the extremities of people with Parkinson's disease [1]
Harrigan et al*.,*[13,12]	Written score sheets of clinical assessments [1] Output from instrumentation attached to participants [8], [9], [10], [11], [12] Signals from instrumentation [12] Fast Fourier transforms of signals [12] Amor and bump continuous wavelet transforms of signals [12]	Documentation of a protocol [1] administered to participants with Parkinson's disease (*N* = 20), multiple system atrophy-parkinsonian type (*N* = 1), and age- and sex- matched healthy participants with typical development (*N* = 8)
Elshourbagy et al*.,*[23], [24], [25]	Videotapes of clinical assessments [1]	Recording of a protocol [[1], [2]] administered to participants with Parkinson's disease (*N* = 2) and age- and sex- matched healthy participants with typical development (*N* = 3)
Ziegelman et al*.,* 2023 [15,16]	Written descriptions of structure of online ratings [17] Signals from instrumentation [12] Fast Fourier transforms of signals [12] Amor and bump continuous wavelet transforms of signals [12]	Written descriptions and schematic diagrams of electronic files of signals and fast Fourier and amor and bump continuous wavelet transforms presented randomly and blindly to 35 trained raters for independent scoring [17]
Suresh et al*.,*[26]	Classification of transform [12,13] images	Images of transforms of a protocol [1] administered to participants with Parkinson's disease (*N* = 20), multiple system atrophy-parkinsonian type (*N* = 1), and age- and sex-matched healthy participants with typical development (*N* = 8) classified through deep learning into those with low and high impairments [26]

A low-cost quantitative continuous measurement of movements in the extremities of people with Parkinson's disease [1] was administered [2] by raters certified in the Movement Disorder Society-sponsored revision of the Unified Parkinson's Disease Rating Scale (MDS-UPDRS) [22] to cohorts of participants with Parkinson's disease (*N* = 20) and multiple system atrophy-parkinsonian type (*N* = 1) and to age- and sex-matched healthy participants with typical development (*N* = 8). The data were collected utilizing the unique variables in the protocol [1]. Thus, this article is remarkable because the novel variables described in the original protocol [1] are utilized. A retest administration of the protocol [1,2] was performed a month or later after the original administration for participants with Parkinson's disease (*N* = 10) and typical development (*N* = 8). The trained rater who administered the protocol recorded the scores of each administration immediately after each task was performed. Copies of the original score sheets recorded during the live ratings are published [12,13]. The original live administrations of the protocols were videotaped. The videotapes of the original administrations have been published [23], [24], [25]. Validation of the original live ratings has been accomplished by trained raters who scored videos of the original live administrations of the protocol [21].

The data include the electronic online frameworks to represent random images of signals and transforms for virtual rating by experts unfamiliar with the source of the images. The data include tables of the clinical scores by a trained rater for each item of the protocol [1,2] and for each testing session of each participant [12], the electronic frameworks [15,16] randomly representing the signals and transforms of the live ratings and the scores of 35 trained raters [15], [16], [17], [18], [19], [20].

## 4 Experimental Design, Materials, and Methods

A low-cost quantitative structured office measurement of movements in the extremities of people with Parkinson's disease [1] was administered [2] by raters who were certified in the Movement Disorders Society-sponsored revision of the Unified Parkinson's Disease Rating Scale (MDS-UPDRS) [21] to cohorts of (A) adults with Parkinson disease (*N* = 20) and (B) adults with multiple system atrophy-parkinsonian type (*N* = 1) as well as to (C) healthy adults with typical development (*N* = 8). All participants underwent a single test session. Retest sessions a month or later after the initial test were administered to cohorts with Parkinson's disease (*N* = 10) and typical development (*N* = 8). The twelve tasks of the low-cost quantitative structured office measurement of movements in the extremities of people with Parkinson's disease [1,2] were conducted for each assessment.

Data was acquired with a commercial data acquisition system [10]. Small, low power, 3-axis ± 3 g accelerometers [8] mounted on three-axis accelerometer evaluation boards [9] were taped (A) to the second (middle) phalanx of each index finger and midway between the radius and the ulna two inches proximal to the wrist joint on the dorsal surface of each arm gor the tasks of the upper extremities and (B) to the anterior surface of each tibia two inches proximal to the medial malleolus and to the dorsal surface of the proximal phalanx of each big toe for the tasks of the lower extremities [1]. Accelerometers acquired data with a sample rate of 80 Hz. To ensure simultaneous data acquisition from synchronized sensors, four accelerometers (two on each upper extremity for acquisitions from the upper extremities or two on each lower extremity for acquisitions from the lower extremities) were connected to a 16-channel sequential acquisition data logger. Tasks varied from 10 sec to 3 min according to the protocol [1]. The technologist operating the instrumentation began to record the output a few seconds before asking the examiner to begin each task. After each task was completed, the examiner asked the technologist to stop the data acquisition. Output from the data logger [10] was saved in a laptop computer [7]. The output from the data logger [10] was processed to signals with a commercial program in a laptop computer [7]. The signals were then stored in the appropriate folders in the laptop computer for future analysis. The coding forms with the scores of the live ratings [12] and the codes [13] to generate the signals and their transforms have been published [12,13].

The signals and transforms of the five repetitive actions (3.4 Finger tapping, 3.5 Hand movements, 3.6 Pronation-supination movements of hands, 3.7 Toe tapping, and 3.8 Leg agility) [1,2] were expressed as panels constructed to allow a balanced comparison for levels of impairment [15], [16], [17], [18], [19], [20]. The raters were all certified in the MDS-UPDRS [22]. They were instructed to apply the rating system for live in person evaluations of people performing the tasks of the MDS-UPDRS [22] to the signals and transforms generated by the instrumentation for this protocol. The raters were instructed to rate the abnormalities (interruptions, slowing, amplitude decrements) observed in the representations of the signals and transforms with a scale like the scale for live ratings of participants (0 normal, 1 minimal, 2 mild, 3 moderate, 4 worse) [[1], [2], [17], [20], [21], [22]] as follows:

A. Interruptions or freezing

1a. 1 to 2 interruptions
2a. 3 to 5 interruptions
3a. 5 or more interruptions or a freeze, a sustained absence of repetitions
4a. Severe

B. Slowing

1b. Slight
2b. Mild
3b. Moderate
4b. Severe

C. Amplitude reductions

1c. End of sequence
2c. Middle of sequence
3c. Beginning of sequence
4c. Worse

**Fig. 1:**
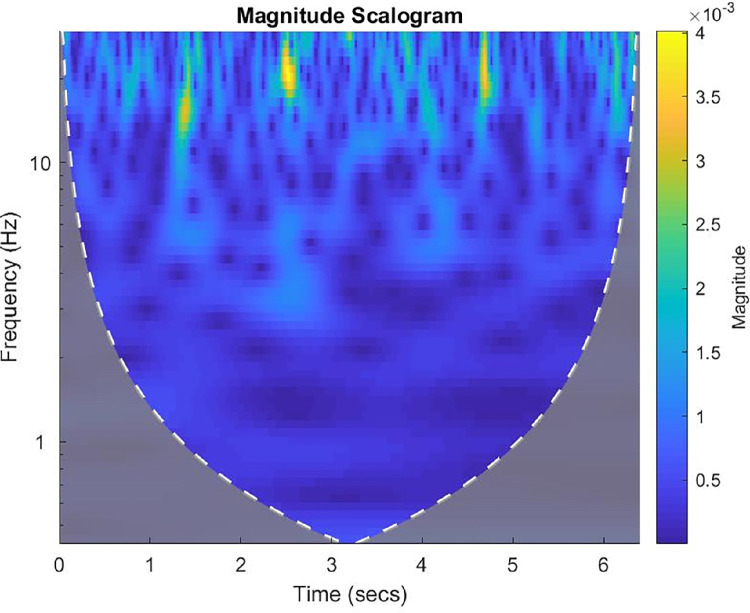
The image of the amor continuous wavelet transform of the x-axis of the accelerometer on the middle phalanx of the left index finger during the performance of the task for 3.5 Hand movements [1,12,13] of a 54-year-old man with multiple system atrophy-parkinsonian type that was presented randomly and blindly to trained raters for scoring to obtain the dataset for this article [12]. There are many interruptions throughout the sequence to merit a score of 3a. The image is so bizarre that scoring for slowing and amplitude decrements cannot be readily assessed. Therefore, the images merit scores of 4b. Severe for Slowing and 4c. Severe for Amplitude reductions. The overall score is 4 because it equals the highest subscores (4b. and 4c.). Reproduced with permission [12].

After attaining certification in the MDS-UPDRS [22], raters were asked to assess two separate random presentations of the images of a man with multiple system atrophy to become familiar with the representation of images. Figure 1 illustrates the image of the continuous wavelet transform of the x-axis of the accelerometer on the middle phalanx of the left index finger during the performance of the task for 3.5 Hand movements [1,12,13]. After completing the scoring of the images of the man with multiple system atrophy, raters were asked to rate the images of the ten participants with Parkinson's disease who underwent a single session followed by a second presentation of the same items in a random order [15], [16], [17], [18], [19], [20]. The raters were then asked to rate two separate random presentations of the images of signals and transforms of the participants with Parkinson's disease (*N* = 10) and healthy age- and sex-matched participants with typical development (*N* = 8) who underwent two rating sessions for test and retest [15], [16], [17], [18], [19], [20].

Accelerometry signals were visually observed by the trained raters to identify deviations from regular rhythms. Interruptions and halts in the signals were identified by the absence of the regular cycle. Slowing of cycles was identified by a longer duration between the signals. Amplitude decrements were identified by reductions in the height of the peaks and the depths of the troughs in the cycles. There were variations in the signals for participants with Parkinson's disease and multiple system atrophy-parkinsonian type and for the healthy participants with typical development. The rating system could readily be applied to the output of individuals with mild or moderate impairments. When the signals of individuals with severe impairments did not match the rating system, a score of 4 was given. Several individuals with severe impairments could not perform the tasks generating absent or bizarre signals. These participants had output that attained the floor effect of the rating system. In other words, the output was so abnormal that the rating system for normal, slight, mild, or moderate did not match. This rating procedure does not adequately capture the motor skills of people with severe impairments. This rating procedure is appropriate for people without impairments or with slight, mild, or moderate impairments. The current study demonstrates that this process is feasible. Future studies are needed to establish the validity of this approach with other populations of participants and raters by colleagues in other locations.

**Fig. 2:**
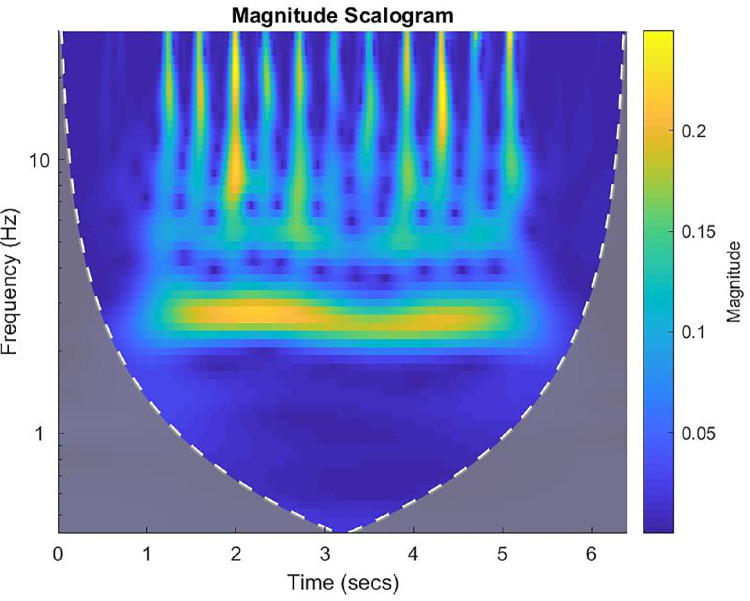
The image of the amor continuous wavelet transform of the y-axis of the accelerometer on the proximal of the left big toe during the performance of the task for 3.7 Toe tapping [1,12,13,] of a 57-year-old man with Parkinson's disease that was presented randomly and blindly to trained raters for scoring to obtain the dataset for this article [12]. The frequency is consistent at approximately 5 Hz. The absence of interruptions and freezes is normal. The frequency is slightly reduced during 3 to 5 s. to merit a score of 1b. The amplitude decreases between 3 to 4 s as indicated by the reduced orange brightness. The reduction of amplitude in the middle of the sequence merits a score of 2c. The overall score is 2 representing the highest abnormal score, 2c. Reproduced with permission [12].

Examples of the interruptions, freezes, slowing, and amplitude decrements identified by visual inspection of continuous wavelet transforms are presented in Figures 1 and 2.

The protocol for collecting/creating the data has been published to be utilized by all in the research community [1,17,21]. The videos of the sessions have been published [23], [24], [25] and analyzed [21].

We are pleased to freely provide all raw data as well as the protocols to generate the data and all videos, tables, graphs, images, charts, and analyses of the data [1,2,12,13,[15], [16], [17], [18], [19], [20], [21],[23], [24], [25], [26], [27]]. Additionally, we shall continue to present and publish the future investigations building on these data to shall freely with the research community our inventions.

## 5 Limitations

There were many limitations of this preliminary investigation [13,17].

The data for this pilot investigation did not rigorously follow a scientific method employing optimal sampling methods representative of the population. Instead, a convenience sample of participants who attended the outpatient clinics of the collaborating neurologists was employed for the cohorts with Parkinson's disease and multiple system atrophy. Similarly, a convenience sample of colleagues and friends generated the sample of healthy age- and sex-matched participants with typical development. Since there was no funding for this investigation, no compensation was provided for the participants. All the participants volunteered to take part in the investigation without renumeration. Additionally, the trained raters were recruited from friends and colleagues without compensation. The cohort of trained raters constitutes a convenience sample of those individuals who agreed to take part in the study as volunteers. Future investigation will utilize a rigorous sampling method for future investigations [28]. Although no questionnaire/survey was employed for this study, we are developing direct, unambiguous, and unbiased tools for future investigations.

Another limitation of this study was the variability of the scores of the raters. Although all raters had attained certification in the MDS-UPDRS [22], the gold-standard structured assessment of Parkinson's disease, the current project represented the initial use of the current protocol [17] for online ratings of signal and transforms by visual observation as a team. There were differences in the ratings obtained by the raters on the initial viewing of the images. Although all raters used the same electronic framework to view the images, there may have been differences in the representations on the monitors of each rater. All raters used different monitors in different locations resulting in differences among the images seen by raters. Internet disconnections interfered with some rating sessions. Additionally, there may have been subjective and other biases affecting the ratings by the individuals. Raters were asked to apply the rating scale as best they could even though the images may be challenging to score with the given scale [17]. Visual inspection of the scores of each item by individual raters [15] demonstrates considerable variability. Assessment of the differences among the scores in this enormous data set [15] is an ongoing project.

Data from the prior ratings for all prior studies have been published and are readily available to all [16,17]. We are formulating mechanisms to provide to the research community the means to rate the extant images of signals and transforms for comparison with the scores of the trained raters who have already rated all items [16,17].

## 6 Future Directions

Future studies will include multiple investigators for simultaneous collection, rating, and analysis of components of the motor assessment procedures. Thus, interrater reliability will be assessed throughout each stage of the experiemental protocol. Additionally, future studies will compare and contrast the output of live and videotaped motor assessments to evaluate concurrent validity. The results from the original data and the analyzed data will be assessed for convergent validity.

We are developing the means for colleagues around the world to submit signals and transforms from their own rhythmic processes into the existing electronic framework. We are formulating the means for colleagues to rate their own submitted signals and transforms and the means for trained raters to rate those signals and transforms. Thus, our colleagues will rate their signals and transforms and additionally obtain scores of ratings of their signals and transforms from trained raters. Colleagues will then learn the rating process and will receive the scores of trained raters for comparison and contrast. Interpretation of the scores will also be provided to those who submit signals and transforms. Colleagues will utilize the data in this article and the protocols to generate and analyze the data as templates for their own data. Thus, this collection of data constitutes a valuable tool for the research community to utilize for the generation, recording, and analysis of their own investigations. The means to generate and analyze the data in this article is available through open access for the use of the research community at no cost. We are pleased to share our inventions with all for beneficial purposes.

We are developing methods to apply machine learning for the rating of signals and transforms. Thus, automatic intelligence will generate ratings of signals and transforms for comparison and contrast with the collection of scores by visual observation by trained raters. We anticipate that the automatic classification procedures will generate the means to distinguish signals and transforms of people with Parkinson's disease from those of parkinsonisms, syndromes with some clinical features of Parkinson's disease and different clinical courses and prognoses. Additionally, we anticipate that the current data can be developed to distinguish Parkinson's disease and other involuntary movement disorders from voluntary movements including *zaghrouta* and other expressions of emotion, factitious movement disorders, and malingering [29,30].

We seek to share this dataset with the research community at this time because colleagues can then utilize this template to analyze and interpret their data collected in the past and in the future. The dataset can be used as a template to generate and analyze both unsupervised and supervised machine learning algorithms [26,27]. Thus, the procedures described in this article will provide the foundation to enhance the analysis many data sets including characteristics of the gait of stallions at walk and at trot [31], the finger tapping by healthy young adults with typical development [32], and the locomotor activity of rats [33]. We anticipate that the procedures described in this article will facilitate the analysis and interpretation of a vast spectrum of datasets of rhythmic processes. For this reason, we seek to publish this article at this time.

## Ethics Statements

The work described has been carried out in accordance with the Code of Ethics of the World Medical Association (Declaration of Helsinki) for experiments involving humans [34]. The manuscript utilizes the Recommendations for the Conduct, Reporting, Editing and Publication of Scholarly Work in Medical Journals and includes representative human populations (sex, age, and ethnicity) as per those recommendations [35]. The terms sex and gender are used correctly.

The data was collected according to the guidelines of the Declaration of Helsinki [34] and approved by the Institutional Review Board of the Johns Hopkins School of Medicine in Baltimore, Maryland (Protocol Number: IRB00110166 and Initial Approval Date: 22 September 2016). Written informed consent was obtained from each participant.

## CRediT authorship contribution statement

**Liran Ziegelman:** Conceptualization, Methodology, Software, Validation, Formal analysis, Investigation, Resources, Data curation, Writing – review & editing, Visualization, Supervision, Project administration. **Tanvi Kosuri:** Conceptualization, Methodology, Software, Validation, Formal analysis, Investigation, Resources, Data curation, Writing – review & editing, Visualization, Supervision, Project administration. **Husain Hakim:** Methodology, Software, Validation, Formal analysis, Investigation, Resources, Data curation. **Luqi Zhao:** Methodology, Resources, Data curation. **Abdelwahab Elshourbagy:** Methodology, Resources, Data curation. **Kelly Alexander Mills:** Methodology, Validation, Formal analysis, Investigation, Resources, Data curation. **Timothy Patrick Harrigan:** Conceptualization, Methodology, Software, Validation, Formal analysis, Investigation, Resources, Data curation. **Manuel Enrique Hernandez:** Conceptualization, Methodology, Software, Validation, Formal analysis, Investigation, Resources, Data curation, Writing – review & editing, Visualization, Supervision, Project administration. **James Robert Brašić:** Conceptualization, Methodology, Software, Validation, Formal analysis, Investigation, Resources, Data curation, Writing – review & editing, Visualization, Supervision, Project administration.

## Declaration of Competing Interests

The authors declare that they have no known competing financial interests or personal relationships that could have appeared to influence the work reported in this paper.

## Data Availability

Classification of extremity movements by visual observation of signal transforms (Original data) (Mendeley Data)
